# Molecular Docking Analysis of Selected Urtica dioica Constituents As Human Carbonic Anhydrase II (hCA-II), Human 11 Beta-Hydroxysteroid Dehydrogenases Type 1 (h11beta-HSD1), and Human Dual Specificity Phosphatase (hCDC25B) Inhibitory Agents

**DOI:** 10.7759/cureus.53886

**Published:** 2024-02-08

**Authors:** Angel Jenifer Arulselvan, Mani Sankar Manimuthu, Radhakrishnan Narayanaswamy

**Affiliations:** 1 Biochemistry, Saveetha Medical College and Hospital, Saveetha Institute of Medical and Technical Sciences (Deemed to be University), Chennai, IND

**Keywords:** stinging nettle, human dual specificity phosphatase (hcdc25b), human 11 beta-hydroxysteroid dehydrogenases type 1 (h11beta-hsd1), human carbonic anhydrase ii (hca-ii), docking, good health and well-being, urtica dioica

## Abstract

Background

*Urtica dioica* (Stinging nettle)has been reported to exhibit various pharmacological activities. In the present study, we aimed to evaluate 24 selected constituents of *U. dioica* as potent inhibitory agents of human carbonic anhydrase II (hCA-II), human 11 beta-hydroxysteroid dehydrogenases type 1 (h11beta-HSD1), and human dual specificity phosphatase (hCDC25B) using *in silico* method.

Methodology

The 24 selected constituents of *U. dioica* (Stinging nettle) were studied on the docking behavior of hCA-II, h11beta-HSD1, and hCDC25B by using the Webina docking method. In addition to docking, toxicity analysis was also performed using the pkCSM free web server, respectively.

Results

Toxicity analysis has shown that six ligands (25%) of *U. dioica *(Stinging nettle) are predicted to have hERG II (Human ether-a-go-go-related gene) inhibition activity. The docking analysis showed that afzelin, stigmastane-3, 6-diol, and astragalin of *U. dioica* have shown the maximum binding energy (-7.2, -9.5, and -8.5 kcal/mol) with the hCA-II, h11beta-HSD1 and hCDC25B, respectively.

Conclusions

Thus, the current finding provides new knowledge about the 24 selected ligands of *U. dioica* (Stinging nettle) as potent inhibitory agents of human carbonic anhydrase II (hCA-II), human 11 beta-hydroxysteroid dehydrogenases type 1 (h11beta-HSD1) and human dual specificity phosphatase (hCDC25B).

## Introduction

Carbonic anhydrase II (CA II) is one of the metalloenzymes that catalases the reversible hydration of carbon dioxide into a bicarbonate ion. It participates in a variety of physiological functions, such as bone reabsorption, respiration, and acid-base balance [1]. CA II deficiency syndrome [a human autosomal recessive disorder] is characterized by i) cerebral calcification, ii) osteopetrosis, and iii) renal tubular acidosis [1]. Studies on naturally occurring phytochemicals that inhibit carbonic anhydrase II activity have been reported to possess therapeutic uses. Similarly, the metabolism of glucocorticoids is catalyzed by two enzymes namely 11 beta-hydroxysteroid dehydrogenases type 1 and 2 (11 beta-HSD1 and 11 beta-HSD2). These enzymes are involved in the inter-conversion of the non-receptor binding inactive form (cortisone) and the receptor binding active form (cortisol). 11 beta-HSD1 is an NADP(H)-the dependent enzyme that converts inactive cortisone to active cortisol in the adipose tissue, brain, liver, and vasculature [2]. An excessive amount of 11 beta-HSD1 enzyme activity has been reported to be involved in metabolic syndrome and its associated cardiovascular complications [2]. Therefore, identifying natural substances that inhibit 11 beta-HSD1 activity has been reported to possess therapeutic applications. Another key enzyme namely, dual specificity phosphatases (DUSP) [CDC25A, CDC25B and CDC25C] are the enzymes that regulate phosphorylation of cyclin-dependent kinases (substrates) during the cell cycle progression. In contrast, (i) cancer, (ii) diabetes, and (iii) neurodegenerative disorders have been associated with dysregulation of DUSP activity [3]. Therefore, identifying natural compounds that inhibit DUSP activity has been reported to possess therapeutically uses.

*Urtica dioica* (Stinging nettle) belongs to the family Urticaceae, a medicinal perennial flowering plant, commonly referred as “stinging nettle” and in Tamil, it is referred as “Peru-n-kanchori (or) Punnai kasarai.” *Urtica *species have been used in treatment of rheumatism, sciatica, dandruff, coughs, diabetes, diarrhoea, eczema, gout, fever, haemorrhoids, nosebleeds, scurvy, snake bites, allergies, arthritis, and urinary tract infections [4]. It has also been established that its extracts can successfully reduce inflammation in osteoarthritis, allergic rhinitis, and prostatitis [5-7]. *U. dioica* has been demonstrated to be an effective treatment for benign prostatic hyperplasia [8,9]. Studies indicate that *U. dioica* extracts may be useful in lowering symptoms including sneezing, itching, nasal congestion, and allergic rhinitis [10]. In diabetic rats, *U. dioica* extract successfully lowered blood glucose levels [11]. Extracts from *U. dioica* showed excellent antioxidant, anti-inflammatory, antiulcer, antiviral, anticancer, antibacterial, antifungal, and insecticide properties [12]. Thus the earlier investigations prompted us to carry out the current study on twenty four selected *Urtica dioica* constituents which includes afzelin, 2-aminooctadecane-1,3,4-triol, astragalin, 1,2,3-butanetriol, carvacrol, carvone, cycloolivil, estra-1,3,5-trien-ol, ethyl-iso allochalate, kephton, neoolivil, 14-octacosanol, Oxime-methoxy-phenyl, pinanediol, pinene-9,10-diol, pinoresinol, scoparone, 6,6-dimethyl-2-methylenebicyclo [3.1.1] heptane, 1-[3,4-dihydroxyphenyl]-1,2, 4-(3-hydroxyphenyl), 3-[4-hydroxyphenyl], 3-hydroxyacetophenone, stigmastane-3,6-diol and 5,6,7,8-tetrahydroxy-2-(4-hydroxyphenyl)chroman-4-one. These above said *Urtica dioica* (Stinging nettle) constituents were aimed to study on the docking analysis of human carbonic anhydrase II (hCA-II), human 11 beta-hydroxysteroid dehydrogenases type 1 (h11 beta-HSD1) and human dual specificity phosphatase (hCDC25B) by employing the Webina docking method. Furthermore, toxicity analysis was also studied using pkCSM free web server.

## Materials and methods

Ligand preparation

Chemical structures of the 24 *U. dioica* (Stinging nettle) ligands namely 1) Afzelin (PubChem ID 5316673); 2) 2-Aminooctadecane-1,3,4-triol (PubChem ID 248575); 3) Astragalin (PubChem ID 5282102); 4) 1,2,3-Butanetriol (PubChem ID 20497); 5) Carvacrol (PubChem ID 10364); 6) Carvone (PubChem ID 7439); 7) Cycloolivil (PubChem ID 5316262); 8) Estra-1,3,5(10)-trien-17B-ol; 9) Ethyl iso- allocholate; 10) Kephton (PubChem ID 5280483); 11) Neoolivil (PubChem ID 9976812); 12) 14-Octacosanol (PubChem ID 5320252); 13) Oxime- methoxy-phenyl; 14) 9, 10-Pinanediol; 15) Pinene-9,10- diol; 16) Pinoresinol (PubChem ID 73399); 17) Scoparone (PubChem ID 8417); 18) 6,6-dimethyl-2-methylenebicyclo[3.1.1] heptane; 19) 1-(3,4-Dihydroxyphenyl)-1,2-propanediol; 20) 4-(3-hydroxypropyl)-2-Methoxyphenol; 21) 3-[4-Hydroxyphenyl]-2-propen-1-ol; 22) 3-Hydroxyacetophenone; 23) Stigmastane-3, 6-diol (PubChem ID 12070151); 24) 5,6,7,8-tetrahydroxy-2-(4-hydroxyphenyl)chroman-4-one were retrieved from PubChem compound database. Unavailable three-dimensional structures were drawn using ChemSketch. Ligands were prepared using ChemDraw 2D and 3D [13]. Thus, these prepared three-dimensional structures were used for further study (Webina docking).

Toxicity analysis

Toxicity analysis was determined for 24 selected ligands of *U. dioica* (Stinging nettle) using the pkCSM (predicting small-molecule pharmacokinetic properties using graph-based signatures method) free web server [14].

Identification and preparation of target enzyme

The three-dimensional (3D) structure of human carbonic anhydrase II (hCA-II) (PDB ID: 1BCD with a resolution of 1.90 Aᵒ), human 11 beta-hydroxysteroid dehydrogenases type 1 (h11 beta-HSD1) (PDB ID: 2BEL with a resolution of 2.11 Aᵒ) and human dual specificity phosphatase (hCDC25B) (PDB ID: 1QB0 with a resolution of 1.91 Aᵒ) was obtained from Protein Data Bank (PDB). A chain of these enzymes was processed individually by removing other chains, ligands, and crystallographically observed water (H2O) molecules (i.e., water without hydrogen bonds) by utilizing UCSF Chimera software [14].

Docking study

A docking analysis was performed for 24 selected constituents of *U. dioica* (Stinging nettle) using the Webina (runs based on AutoDock Vina method) free web server [15]. Webina docking method is simple and straight forward one, where it allows users to use various file formats namely mol, pdb and sdf to upload both enzymes (receptors) and ligands input files. Similarly, it also allows users to setting up docking calculations (identifying an appropriate docking box) and analyzing docking output (examining/virtualizing predicted binding poses).

## Results

Toxicity analysis has shown that all the ligands of *U. dioica* do not exhibit any hERG I (human ether-a-go-go-related gene-I) inhibition activity (Table 1).

**Table 1:** Toxicity analysis of 24 Urtica dioica (Stinging nettle) ligands using the pkCSM free web server. Note: AMES● -AMES toxicity, hERG I■- Human ether-a-go-go-related gene inhibitor I, hERG II□- Human ether-a-go-go-related gene inhibitor II, ORAT◌- Oral rat acute toxicity (Lethal dose LD50 in mol/kg), HT◊ - Hepatotoxicity, SS*- Skin sensitisation, MT**- Minnow toxicity (log mM).

Ligand name	AMES^●^	hERG I^■^	hERG II^□^	ORAT^◌^	HT^◊^	SS^*^	MT^**^
Afzelin	No	No	Yes	2.61	No	No	3.73
2-Amino Octadecane-1,3,4-triol	No	No	No	1.78	No	Yes	0.40
Astragalin	No	No	No	2.55	No	No	6.74
1,2,3-Butanetriol	No	No	No	1.10	No	No	3.38
Carvacrol	No	No	No	2.07	Yes	Yes	1.21
Carvone	No	No	No	1.86	No	Yes	1.45
Cycloolivil	No	No	Yes	2.38	No	No	3.44
Estra-1,3,5(10)-trien-17B-ol	No	No	Yes	2.70	No	No	0.54
Ethyl iso- allocholate	No	No	No	2.05	No	No	0.34
Kephton	No	No	Yes	1.72	No	No	-5.27
Neoolivil	No	No	No	2.31	No	No	1.78
14-Octacosanol	No	No	Yes	1.84	No	Yes	-3.78
Oxime- methoxy-phenyl	Yes	No	No	2.13	No	No	2.02
9, 10-Pinanediol	No	No	No	1.57	No	Yes	2.15
Pinene-9,10- diol	No	No	No	2.01	No	Yes	1.74
Pinoresinol	No	No	No	2.22	No	No	1.10
Scoparone	No	No	No	2.35	No	No	1.22
6,6-dimethyl-2-methylene	No	No	No	1.70	No	No	1.01
1-[3,4-Dihydroxyphenyl]-1,2-propanediol	Yes	No	No	1.96	No	No	1.90
4-(3-hydroxypropyl)-2- methoxyphenol	No	No	No	1.95	No	Yes	1.93
3-[4- Hydroxypheny l]-2-propen-1-ol	No	No	No	1.80	No	Yes	1.71
3- Hydroxyacetophenone	No	No	No	1.87	No	No	1.69
Stigmastane-3, 6-diol	No	No	Yes	2.81	Yes	No	-0.98
5,6,7,8-tetrahydroxy-2-(4- hydroxyphenyl)chroman-4-one	Yes	No	No	2.30	No	No	1.86

Two ligands (carvacrol and stigmastane-3, 6-diol) of *U. dioica* (Stinging nettle) have been predicted to exhibit a hepatotoxicity nature (Table 1). Similarly, seven ligands (2-amino octadecane-1, 3, 4-triol, carvacrol, carvone, 14-octacosanol, 9, 10-pinanediol, pinene-9, 10-diol, 4-(3-hydroxypropyl)-2-methoxyphenyl and 3-[4-hydroxypheny l]-2-propen-1-ol]) of *U. dioica* (Stinging nettle) have predicted to shown skin sensitization effect.

The docking analysis showed that afzelin exhibited the maximum binding energy (-7.2 kcal/mol) with the human carbonic anhydrase II (hCA-II) enzyme. In contrast, 14-octacosanol exhibited the least binding energy (-4.1 kcal/mol) with the hCA-II enzyme (Table 2). The binding energy analysis of the present study showed the hCA-II in the following order: afzelin (-7.2 kcal/mol), ˂ estra-1,3,5(10)-trien-17B-ol, ethyl iso- allocholate, pinoresinol (-7.1 kcal/mol), ˂ astragalin (-6.9 kcal/mol), ˂ neoolivil, stigmastane-3, 6-diol (-6.7 kcal/mol), ˂ cycloolivil, kephton, 5,6,7,8-tetrahydroxy-2-(4-hydroxyphenyl)chroman-4-one (-6.6 kcal/mol), ˂ 1-[3,4-dihydroxyphenyl]-1,2-propanediol (-6.4 kcal/mol), ˂ scoparone (-6.0 kcal/mol), ˂ carvone (-5.8 kcal/mol), ˂ oxime- methoxy-phenyl (-5.7 kcal/mol), ˂ 3-[4- hydroxypheny l]-2-propen-1-ol, 3- hydroxyacetophenone (-5.6 kcal/mol), ˂ 9, 10-pinanediol (-5.5 kcal/mol), ˂ 4-(3-hydroxypropyl)-2-methoxyphenol (-5.4 kcal/mol), ˂ pinene-9,10- diol (-5.2 kcal/mol), ˂ 2-amino Octadecane-1,3,4-triol (-5.1 kcal/mol), ˂ 6,6-dimethyl-2-methylene (-4.8 kcal/mol), ˂ 1,2,3-butanetriol (-4.4 kcal/mol) and ˂ 14-octacosanol (-4.1 kcal/mol). Similarly, docking analysis revealed that stigmastane-3, 6-diol exhibited the maximum binding energy (-9.5 kcal/mol) with the human 11 beta-hydroxysteroid dehydrogenases type 1 (h11 beta-HSD1) enzyme. In contrast, 1, 2, and 3-butanetriol exhibited the least binding energy (-4.3 kcal/mol) with the h11 beta-HSD1 enzyme (Table 2). The binding energy analysis of the current study showed the h11 beta-HSD1 enzyme in the following order: stigmastane-3, 6-diol (-9.5 kcal/mol), ˂ ethyl iso- allocholate (-9.3 kcal/mol), ˂ estra-1,3,5(10)-trien-17B-ol (-9.1 kcal/mol), ˂ afzelin, astragalin (-9.0 kcal/mol), ˂ pinoresinol (-8.5 kcal/mol), ˂ kephton, neoolivil (-8.1 kcal/mol), ˂ 5,6,7,8-tetrahydroxy-2-(4-hydroxyphenyl)chroman-4-one (-8.0 kcal/mol), ˂ cycloolivil (-7.2 kcal/mol), ˂ scoparone (-6.3 kcal/mol), ˂ 1-[3,4-dihydroxyphenyl]-1,2-propanediol (-6.1 kcal/mol), ˂2-Amino Octadecane-1,3,4-triol, carvacrol, 14-octacosanol (-5.7 kcal/mol), ˂ carvone, pinene-9,10- diol, 4-(3-hydroxypropyl)-2-methoxyphenol, 3-[4- hydroxypheny l]-2-propen-1-ol (5.6 kcal/mol), ˂ 3- hydroxyacetophenone (-5.4 kcal/mol), ˂ 6,6-dimethyl-2-methylene (-5.2 kcal/mol) and ˂ 1,2,3-butanetriol (-4.3 kcal/mol).

**Table 2:** The binding energy analysis of 24 selected Urtica dioica (Stinging nettle) ligands with the human carbonic anhydrase II (hCA-II), human 11 beta-hydroxysteroid dehydrogenases type 1 (h11 beta-HSD1) and human dual specificity phosphatase (hCDC25B) enzyme using the Webina docking method.

Ligand name	hCA-II	h11 beta-HSD1	hCDC25B
Binding energy (-kcal/mol)			
Afzelin	-7.2	-9	-8.1
2-Amino Octadecane-1,3,4-triol	-5.1	-5.7	-7.9
Astragalin	-6.9	-9	-8.5
1,2,3-Butanetriol	-4.4	-4.3	-5.6
Carvacrol	-5.6	-5.7	-6.3
Carvone	-5.8	-5.6	-6.3
Cycloolivil	-6.6	-7.2	-7.3
Estra-1,3,5(10)-trien-17B-ol	-7.1	-9.1	-7.0
Ethyl iso- allocholate	-7.1	-9.3	-7.1
Kephton	-6.6	-8.1	-8.2
Neoolivil	-6.7	-8.1	-8.1
14-Octacosanol	-4.1	-5.7	-7.6
Oxime- methoxy-phenyl	-5.7	-5.2	-6.4
9, 10-Pinanediol	-5.5	-5.2	-6.3
Pinene-9,10- diol	-5.2	-5.6	-6.4
Pinoresinol	-7.1	-8.5	-7.2
Scoparone	-6	-6.3	-6.4
6,6-dimethyl-2-methylene	-4.8	-5.2	-6.1
1-[3,4-Dihydroxyphenyl]-1,2-propanediol	-6.4	-6.1	-6.2
4-(3-hydroxypropyl)-2- methoxyphenol	-5.4	-5.6	-6.6
3-[4- Hydroxypheny l]-2-propen-1-ol	-5.6	-5.6	-6.4
3- Hydroxyacetophenone	-5.6	-5.4	-6.3
Stigmastane-3, 6-diol	-6.7	-9.5	-7.6
5,6,7,8-tetrahydroxy-2-(4- hydroxyphenyl)chroman-4-one	-6.6	-8	-6.8

Furthermore, docking analysis revealed that astragalin exhibited the highest binding energy (-8.5 kcal/mol) with the human dual specificity phosphatase (hCDC25B) enzyme. In contrast, 1, 2, and 3-butanetriol showed the least binding energy (-5.6 kcal/mol) with the hCDC25B (Table 2). The binding energy analysis of the present study showed the hCDC25B enzyme in following order: astragalin (-8.5 kcal/mol), ˂ kephton (-8.2 kcal/mol), ˂ afzelin, neoolivil (-8.1 kcal/mol), ˂ 2-amino octadecane-1,3,4-triol (-7.9 kcal/mol), ˂ 14-octacosanol, stigmastane-3, 6-diol (-7.6 kcal/mol), ˂ cycloolivil (-7.3 kcal/mol), ˂ pinoresinol (-7.2 kcal/mol), ˂ 5,6,7,8-tetrahydroxy-2-(4-hydroxyphenyl)chroman-4-one (-6.8 kcal/mol), ˂ 4-(3-hydroxypropyl)-2- methoxyphenol (-6.6 kcal/mol), ˂ oxime- methoxy-phenyl, pinene-9,10- diol, scoparone, 3-[4- hydroxypheny l]-2-propen-1-ol (-6.4 kcal/mol),˂ carvacrol, carvone, 9, 10-pinanediol, 3- hydroxyacetophenone (-6.3 kcal/mol), ˂ 1-[3,4-dihydroxyphenyl]-1,2-propanediol (-6.2 kcal/mol), ˂ 6,6-dimethyl-2-methylene (-6.1 kcal/mol) and ˂ 1,2,3-Butanetriol (-5.6 kcal/mol).

## Discussion

**Figure 1:**
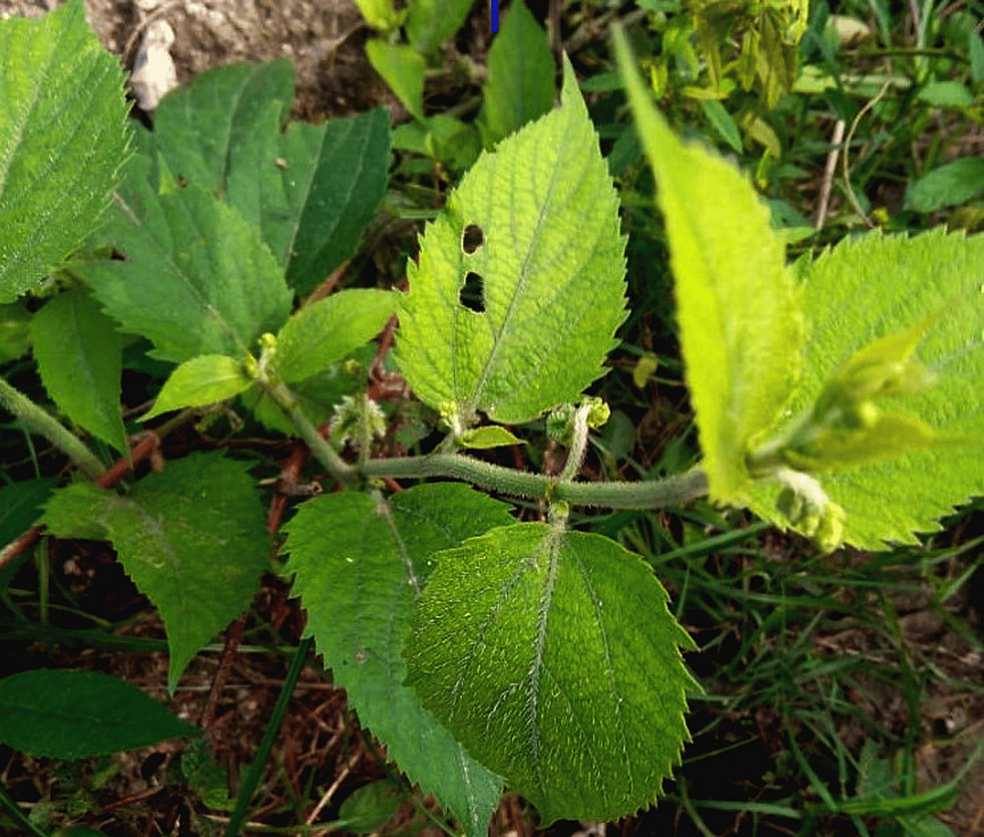
U. dioica (Stinging nettle) plant. Represents the Urtica dioica (Stinging nettle) plant photo taken from Kollar Village, Villupuram District, Tamil Nadu, India, on January 13, 2021.

The phyto-constituents from *U. dioica* (Stinging nettle) have been reported by employing traditional liquid chromatography (LC) separation, high-performance liquid chromatography [16] and ultra-performance liquid chromatography (UPLC) techniques. Moreover, LC-MS [17] and LC-ESI-MS [18] have been used for isolation and chemical characterization. Furthermore, gas chromatography (GC), liquid chromatography (LC) along with mass spectrometry (MS) analytical techniques have been applied for chemical characterization [19]. Numerous reports have demonstrated that *U. dioica* (Stinging nettle) extracts have shown to possess several biological properties such as analgesic, anti-diabetic, anti-cancer, anti-inflammatory, anti-microbial, anti-mutagenic, antioxidant, anti-ulcer, cardiovascular, immune-modulatory activities [20, 21]. *U. dioica* (Stinging nettle) has been known for food applications and has been consumed as green salad, soup, and tea. Chlorophyll extracted on a large scale has been used as a food coloring (green) agent [22]. Figure 1 shows the *U. dioica* (Stinging nettle) plant image. The current study findings are based on *in silico* analysis which provides detailed information about these 24 ligands of *U. dioica* (Stinging nettle) as hCA-II, h11beta-HSD1, and hCDC25B enzyme inhibition activities and are considered as initial research work. Furthermore, in vitro and in vivo experiments are required to confirm these 24 phytoconstituents of *U. dioica* (Stinging nettle) as good enzyme inhibitors against hCA-II, h11beta-HSD1, and hCDC25B activities.

Moreover, Durak et al. reported that the *U. dioica* aqueous extract has been shown to inhibit adenosine deaminase (ADA) activity [23]. Roschek et al. reported that the *U. dioica* aqueous ethanolic extract has shown to inhibit (i) mast cell tryptase, (ii) cyclooxygenases (COX 1 and 2), and (iii) hematopoietic prostaglandin D2 synthase (HPGDS); in addition, it reported to possess both the histamine receptor antagonist and histamine receptor negative agonist activities [5]. Altamimi et al. reported that the *U. dioica* aqueous ethanolic extract has been shown to inhibit (i) maltase, (ii) sucrase, (iii) lactase, and as well as glucose transport inhibitory activities [24]. Thus, in the current investigation, we have chosen the human carbonic anhydrase II (hCA-II), human 11 beta-hydroxysteroid dehydrogenases type 1 (h11 beta-HSD1), and human dual specificity phosphatase (hCDC25B) as the target enzymes. Before carrying out the docking studies, it is important to know the toxicity nature of chosen *U. dioica* (Stinging nettle) ligands in order to avoid drug failure as well as to reduce drug development costs [13].

**Figure 2:**
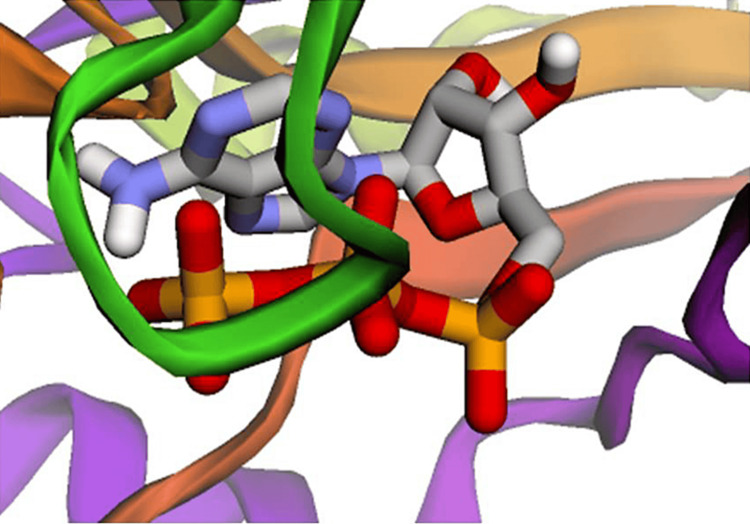
The docking image where afzelin ligand docked with the human dual specificity phosphatase (hCDC25B).

According to International Regulatory Guidelines (namely ICH S7B) all new drugs under the drug development (DD) process should be assessed for effects on the human ether-a-go-go-related gene (hERG) channel prior to clinical trials [25]. In the current investigation, six ligands (afzelin, cycloolivil, estra-1,3,5(10)-trien-17B-ol, kephton, 14-octacosanol and stigmastane-3, 6-diol) of *U. dioica* (Stinging nettle) have predicted to exhibit hERG II inhibition activity, thus six ligands have failed to obey with the above said regulatory guideline (ICH S7B). Gansser and Spiteller (1995) reported for the first time the presence of 14-octacosanol from *U. dioica* (Stinging nettle) root extract and shown to have weak aromatase inhibition activity [26]. Mares and colleagues have reported that inhibitors of human carbonic anhydrase II (hCA-II) have been used for treating glaucoma, neoplasms, and neurodegenerative diseases [27]. The current investigation showed that all 24 ligands of *U. dioica* (Stinging nettle) have docked successfully with the human carbonic anhydrase II (hCA-II) enzyme, which was in good agreement with a recent report [28]. Figure 2 shows the docking image, where the afzelin ligand docked with the human dual specificity phosphatase (hCDC25B).

Hughes et al. reported that inhibitors of human 11 beta-hydroxysteroid dehydrogenases type 1 (h11 beta-HSD1) have been used for treating atherosclerosis, type 2 diabetes mellitus (DM), and metabolic syndrome including obesity [29]. Li et al. reported that inhibitors of human dual specificity phosphatases (DUSP) emerging as a new target in the field of kidney/renal disease [30].

Limitations and future recommendations

The current study findings are based on *in silico* analysis which provides detailed information about these 24 ligands of *U. dioica* (Stinging nettle) as hCA-II, h11beta-HSD1, and hCDC25B enzyme inhibition activities and are considered initial research work. Furthermore, *in vitro* and *in vivo* experiments are required to confirm these 24 phytoconstituents of *U. dioica* (Stinging nettle) as good enzyme inhibitors against hCA-II, h11beta-HSD1, and hCDC25B activities.

## Conclusions

The present study showed that all 24 ligands of *U. dioica* (Stinging nettle) have dock effectively with the three target enzymes namely human carbonic anhydrase II (hCA-II), human 11 beta-hydroxysteroid dehydrogenases type 1 (h11beta-HSD1) and human dual specificity phosphatase (hCDC25B). Interestingly, 1, 2, and 3-butanetriol showed the least binding energy (-4.3 and -5.6 kcal/mol) with both the h11 beta-HSD1 and hCDC25B enzymes, respectively. Thus, the results of the present investigation have shown good knowledge of these 24 ligands of *U. dioica* (Stinging nettle) as potential suppressers against hCA-II, h11beta-HSD1, and hCDC25B concerning the treatments of atherosclerosis, glaucoma, metabolic syndrome (including obesity), neoplasms, neurodegenerative diseases, renal disease, and type 2 DM.
